# Quantitative Palynology Informing Conservation Ecology in the Bohemian/Bavarian Forests of Central Europe

**DOI:** 10.3389/fpls.2017.02268

**Published:** 2018-01-17

**Authors:** Vachel A. Carter, Richard C. Chiverrell, Jennifer L. Clear, Niina Kuosmanen, Alice Moravcová, Miroslav Svoboda, Helena Svobodová-Svitavská, Jacqueline F. N. van Leeuwen, Willem O. van der Knaap, Petr Kuneš

**Affiliations:** ^1^Department of Botany, Faculty of Science, Charles University, Prague, Czechia; ^2^Department of Geography and Planning, University of Liverpool, Liverpool, United Kingdom; ^3^Department of Geography and Environmental Science, Liverpool Hope University, Liverpool, United Kingdom; ^4^Department of Forest Ecology, Faculty of Forestry and Wood Sciences, Czech University of Life Sciences, Prague, Czechia; ^5^Institute of Botany, v.v.i., Czech Academy of Sciences, Průhonice, Czechia; ^6^Institute of Plant Sciences and Oeschger Centre for Climate Change Research, University of Bern, Bern, Switzerland

**Keywords:** biodiversity, Holocene, land-cover, palynology, pollen, REVEALS

## Abstract

In 1927, the first pollen diagram was published from the Bohemian/Bavarian Forest region of Central Europe, providing one of the first qualitative views of the long-term vegetation development in the region. Since then significant methodological advances in quantitative approaches such as pollen influx and pollen-based vegetation models (e.g., Landscape Reconstruction Algorithm, LRA) have contributed to enhance our understanding of temporal and spatial ecology. These types of quantitative reconstructions are fundamental for conservation and restoration ecology because they provide long-term perspectives on ecosystem functioning. In the Bohemian/Bavarian Forests, forest managers have a goal to restore the original forest composition at mid-elevation forests, yet they rely on natural potential vegetation maps that do not take into account long-term vegetation dynamics. Here we reconstruct the Holocene history of forest composition and discuss the implications the LRA has for regional forest management and conservation. Two newly analyzed pollen records from Prášilské jezero and Rachelsee were compared to 10 regional peat bogs/mires and two other regional lakes to reconstruct total land-cover abundance at both the regional- and local-scales. The results demonstrate that spruce has been the dominant canopy cover across the region for the past 9,000 years at both high- (>900 m) and mid-elevations (>700–900 m). At the regional-scale inferred from lake records, spruce has comprised an average of ~50% of the total forest canopy; whereas at the more local-scale at mid-elevations, spruce formed ~59%. Beech established ~6,000 cal. years BP while fir established later around 5,500 cal. years BP. Beech and fir growing at mid-elevations reached a maximum land-cover abundance of 24% and 13% roughly 1,000 years ago. Over the past 500 years spruce has comprised ~47% land-cover, while beech and fir comprised ~8% and <5% at mid-elevations. This approach argues for the “natural” development of spruce and fir locally in zones where the paleoecology indicates the persistence of these species for millennia. Contrasting local and regional reconstructions of forest canopy cover points to a patchwork mosaic with local variability in the dominant taxa. Incorporation of paleoecological data in dialogues about biodiversity and ecosystem management is an approach that has wider utility.

## Introduction

Quantitative reconstructions are fundamental for providing long-term perspectives of ecosystem processes because they can be used to develop baselines for conservation and restoration ecology (National Research Council, 2005; Froyd and Willis, 2008). Paleoecological records have utility in conservation strategies related to biodiversity maintenance, ecosystem naturalness, conservation evaluation, habitat alteration, changing disturbance regimes, and species invasions (e.g., Birks, 1996; Jackson, 1997; Landres et al., 1999; Swetnam et al., 1999; Foster et al., 2003; Gillson and Willis, 2004). Unfortunately, paleoecological research is still largely ignored by conservation biologists and conservationists (Willis and Birks, 2006; Birks, 2012). In 1916, Lennart von Post published the first pollen diagram, providing one of the first qualitative reconstructions of vegetation change that extended over millennial timescales, as well as benchmarking the foundation of palynology (Manten, 1967). Müller (1927) published the first pollen diagram from the Bohemian Forest more than a decade later. Over the past 100 years, palynology has developed from a qualitative tool to a more quantitative analysis of vegetation dynamics that is increasingly well constrained in time (Davis, 2000).

Pollen diagrams produced from mires, peat bogs, and lake sediment profiles, with the data expressed as percentages against time or depth, have been the main way to present data (e.g., Stalling, 1987; Knipping, 1989; Svobodová et al., 2001, 2002; Jankovská, 2006). While percentage pollen diagrams identify changes in vegetation through time, changes in one taxon can affect the percentage proportions of all other taxa. This effect, termed data-closure (Birks and Birks, 1980), can produce disconnections between the trends of pollen and the actual vegetation land-cover. Several quantitative methodologies have been developed to address these issues. Data closure artifacts in pollen percentages can be identified by comparison with pollen influxes (i.e., pollen accumulation rates; grains cm^−2^ yr^−1^), but this requires robust age-to-depth relationships for the sediments. Even then, both percentage and influx data fail to take account of differences in the ecological and environmental factors that influence pollen production and dispersal, nor do they define a spatial scale in reconstructions of vegetation cover (Loidi et al., 2010; Loidi and Fernández-González, 2012). Additionally, because pollen influxes are dependent upon age-to-depth relationships, changes in sedimentation rates can influence pollen concentrations and the subsequent accuracy of influx calculations. Therefore, Sugita (2007a,b) developed the Landscape Reconstruction Algorithm (LRA) which incorporates pollen productivity and pollen dispersal capacity, as well as factors that influence pollen dispersal, such as the size and type of the sedimentation basin. Thus, the LRA provides a more quantitative approach of estimating past vegetation abundance in a defined space. The LRA has two steps: (1) the REgional Vegetation Estimates from Large Sites (REVEALS) model (Sugita, 2007a) estimates pollen-derived regional vegetation cover from large sites (>100 ha) or alternatively many small sites across an area of 10^6^ km^2^ (e.g., Abraham et al., 2014, 2016); and (2) the LOcal Vegetation Estimates (LOVE) model (Sugita, 2007b) uses the regional estimates from REVEALS to estimate pollen-derived local vegetation composition in smaller areas (5–104 ha).

Long-term perspectives on forest dynamics are beneficial for conservation and restoration, and here we demonstrate the utility of quantitative palynological data in the management of Bohemian/Bavarian Forests, including the use of pollen influx data and pollen-landscape reconstruction models (e.g., REVEALS: Sugita, 2007a). Abraham et al. (2016) used the REVEALS model to estimate vegetation cover by integrating pollen sequences from the Šumava region and estimate that spruce has comprised over 50% of the regional forest canopy for the past 7,000 years. Here, we synthesize two newly analyzed, well-dated pollen records from Prášilské jezero (Šumava, Bohemian Forest; Czech Republic) and Rachelsee (Bavarian Forest; Germany) and several previously analyzed pollen records from peat bogs/mires and lakes using multiple quantitative methods to determine the ecological development and vegetation change during the Holocene. The objectives are: (1) to critically evaluate the multiple quantitative approaches used in palynology to reconstruct the history of forest composition; and (2) to explore the utility of quantitative land-cover reconstructions in regional conservation and restoration.

## Background: the bohemian/bavarian forest region of central Europe

**Figure 1:**
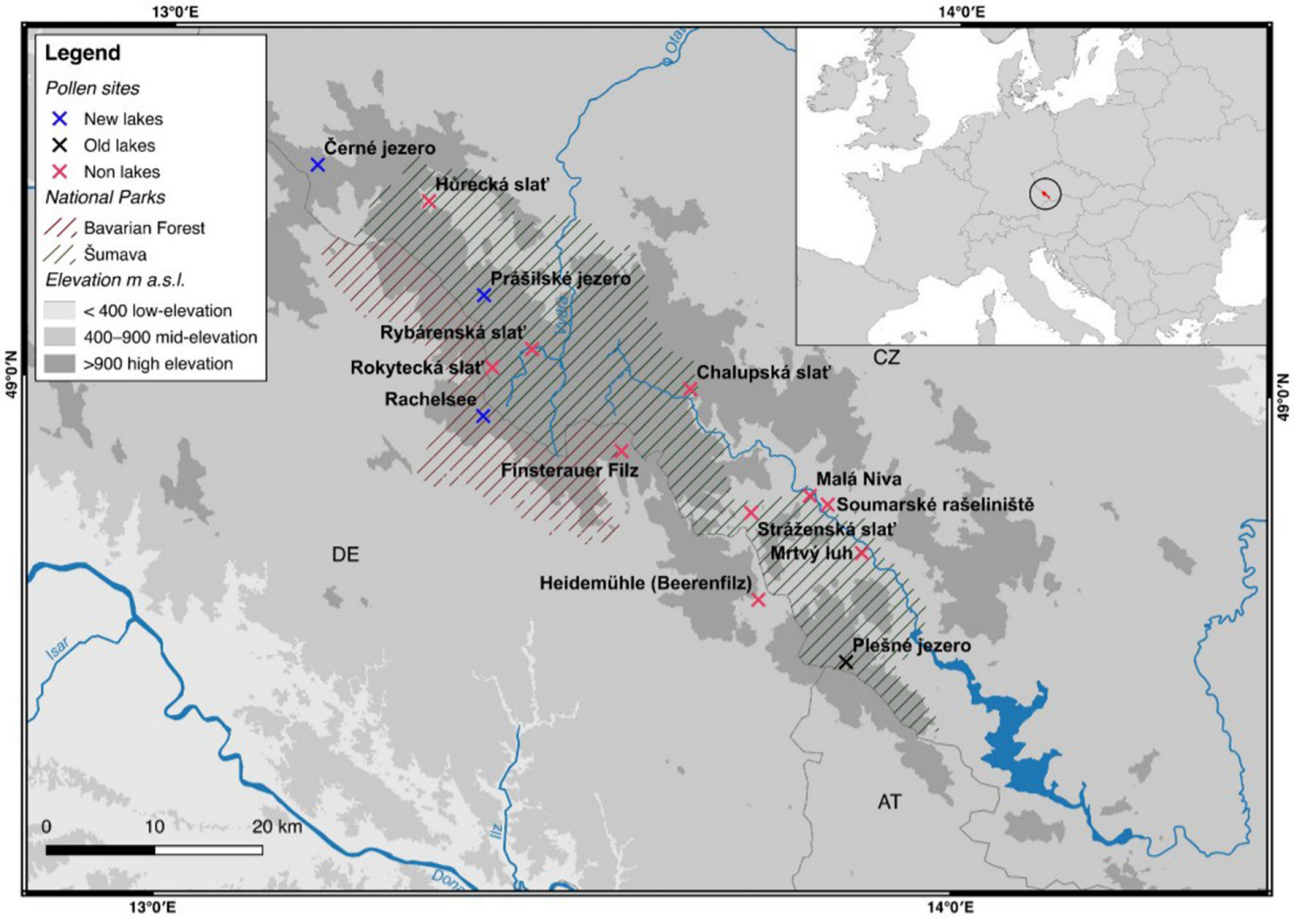
Site map of the Bohemian/Bavarian Forest region of central Europe. Non-lakes (i.e., bogs and/or forest mires) are indicated by a red cross. Newly analyzed lakes presented in this study are indicated by a blue cross. Previously published lakes are indicated by a black cross. The National Park boundaries are indicated by green (Šumava) and red (Bavarian Forest) solid lines.

The Bohemian/Bavarian Forest region of central Europe includes two national parks located along the border of the Czech Republic (Šumava National Park, Bohemian Forest), and Germany (Bavarian Forest National Park^1^, Bavarian Forest; Figure 1). Together, these two parks with their surrounding area comprise one of the largest forested landscapes in central Europe, which is home to many endangered flora (see Křenová and Hruška, 2012) and fauna, and provides a close-to-nature ecosystem of ecological value (Meyer et al., 2009). The Bavarian Forest National Park was established in 1970 with part of Šumava declared a UNESCO Biosphere Reserve in 1990, and Šumava National Park established in 1991.

Subject to long-term human influence, management practices in the Bohemian/Bavarian Forests have ranged from natural development to intensive forest management which has modified both the structure and composition of the forest. Currently, the two parks are divided into wilderness (i.e., not managed), and non-wilderness (i.e., managed). Within the non-wilderness areas, management focuses on sanitary logging of spruce trees killed by previous disturbance events, specifically bark beetle outbreaks. Within Šumava National Park, forest management restoration practices involve reducing spruce populations from the current 84% to a target “natural” representation of 30–40%, and increase beech (ca. 6%) and fir (ca. 1%) up to 35% (Šumava NP Authority). Forest management policies in the Czech Republic direct that the lands between 400 and 900 m altitude should be dominated by beech forests with spruce as a secondary component (Vacek and Mayová, 2000; Pruša, 2001). This management plan is likely based on the Geobotanical Map (Mikyška et al., 1968–1972) and the Map of Potential Natural Vegetation (Neuhäuslová et al., 1998) with beech considered the natural dominant species at mid-elevations in central Europe (Ellenberg and Leuschner, 1996). Within the Bavarian Forest National Park, land managers are concerned with the rapid decline of fir populations in valley bottoms and have focused their attention to factors that could facilitate the natural development of this species (Heurich and Englmaier, 2010), rather than focusing on the removal of spruce which has doubled its range in the Bavarian Forest since the nineteenth century. This strategy is in response to previous paleoecological work documenting that the Bavarian Forests consisted of ~32% fir ~3,000 years ago (Stalling, 1987).

**Figure 2:**
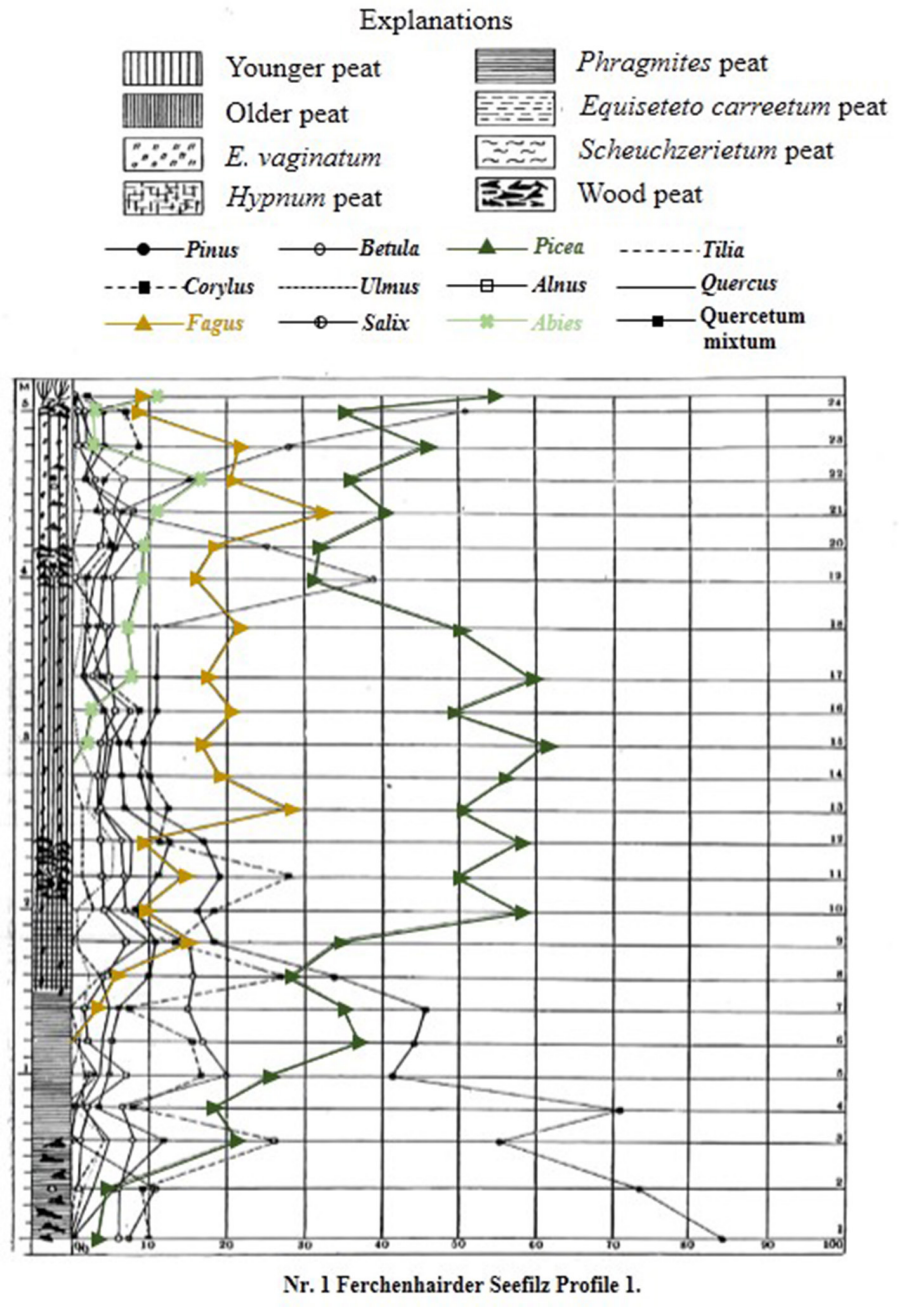
Original pollen percentage diagram of Ferchenhaider Seefilz profile I from the Bohemian/Bavarian Forest region of central Europe. Digitized from Müller (1927), and translated from German into English. Dominant species found in the Bohemian/Bavarian Forests are shown in color; spruce (green line), beech (gold line), and fir (light green line).

Palynological research has a long history in the Bohemian/Bavarian region of central Europe with Müller (1927) publishing one of the first qualitative glimpses of long-term vegetation development in pollen diagrams from several moors in the region (Figure 2). Müller (1927) documented the long-term presence of spruce in the Bohemian/Bavarian Forests, but was unable to discuss these changes relative to time. With widespread application of radiocarbon dating, pollen data from peat sequences (i.e., small peaty wetlands within forests) in Šumava National Park confirm that spruce has dominated the forest canopy cover at elevations >700 m a.s.l. for the past 7,000–8,000 years (Svobodová et al., 2001, 2002). Notwithstanding the better chronological control, the reconstructions presented by Svobodová et al. (2001, 2002) are still limited in that; (1), pollen percentage diagrams offer a qualitative reconstruction of vegetation change through time; and (2) peat bogs/mires and wetlands capture changes in vegetation at various spatial scales [i.e., a forest-stand scale (forest mire) to the extra-local scale (open, wetland mire)]. Reconstructing extra-local to regional-scale vegetation changes is possible by analyzing sediments from lakes as well as open mires, which typically have a much larger pollen catchment area than forest mires. However, pollen-based land-cover models (i.e., REVEALS) are the best approach for reconstructing vegetation abundances at the regional-scale. Currently, the only quantitative land-cover reconstruction of vegetation change in the region using the REVEALS model (Sugita, 2007a) and 14 sediment sequences suggests that spruce forests have comprised ~50% of the forest canopy throughout the past ~7,000 years (Abraham et al., 2016). However, to develop more detailed descriptions of regional-scale vegetation dynamics, further quantitative data are needed. This study provides a more detailed description of land-cover change by analyzing lakes and peat bogs/mires that vary spatially across mid- (700–900 m a.s.l.) and high-elevations (>900 m a.s.l.) from the Bohemian/Bavarian Forests (Table 1), and addresses whether beech has been the dominant forest canopy type at mid-elevations over millennial time-scales.

**Table 1:** Summary of age-depth relationships for Prášilské jezero (Core IDS, PRA-15GC2, PRA 15-2-1, and PRA 15-2-2), Czech Republic and Rachelsee (Core ID, RAA1-4), Germany.

**Depth (cm)**	**Core ID**	**^14^C Age ± 1σb**	**Assigned ^210^Pb (Year AD)**	**Assigned age (cal yr BP)**	**Material dated**	**Lab ID Number**
1,480	PRA 15-2GC		2015 ± 1	−65		Surface
1,480.5	PRA 15-2GC		1995 ± 2	−55		Pb210-1
1,481.5	PRA 15-2GC		1986 ± 3	−36		Pb210-2
1,482.5	PRA 15-2GC		1976 ± 4	−26		Pb210-3
1,483.5	PRA 15-2GC		1963 ± 4	−13		Pb210-4
1,484.5	PRA 15-2GC		1943 ± 5	7		Pb210-5
1,485.5	PRA 15-2GC		1918 ± 7	32		Pb210-6
1,486.5	PRA 15-2GC		1889 ± 9	61		Pb210-7
1,487.5	PRA 15-2GC		1861 ± 12	89		Pb210-8
1,500.5	PRA 15-2-1	590 ± 30			Plant material	Poz-84783
1,539.2	PRA 15-2-1	2, 545 ± 30			Plant material	Poz-81580
1,571.75	PRA 15-2-2	4, 040 ± 35			Plant material	Poz-81582
1,599.75	PRA 15-2-2	5, 700 ± 40			Plant material	Poz-81583
1,628.5	PRA 15-2-1	7, 055 ± 40			Plant material	Poz-87722
1,628.5	PRA 15-2-2	7, 550 ± 40			Plant material	Poz-80182
1,637	PRA 15-2-2	7, 460 ± 40			Plant material	Poz-87724
1,651	PRA 15-2-2	8, 210 ± 50			Plant material	Poz-84781
1,669.5	PRA 15-2-2	9, 330 ± 60			Plant material	Poz-84780
1,690.25	PRA 15-2-2	9, 620 ± 50			Plant material	Poz-80183
57	RAA-1	−62 ± 1				Surface
117	RAA-1	693 ± 30			Plant material	BE-3035
128	RAA-1	1, 170 ± 29			Plant material	BE-3036
147	RAA-1	1, 861 ± 22			Plant material	BE-3037
216	RAA-2	4, 910 ± 35			Plant material	Poz-85119
276	RAA-3	9, 120 ± 50			Plant material	Poz-85121
308	RAA-3	9, 980 ± 60			Plant material	Poz-85122
371	RAA-4	11, 310 ± 40			Organic sediment	Beta-420353

## Methods

### Core retrieval, sediment limnology, and radiocarbon dating

**Figure 3:**
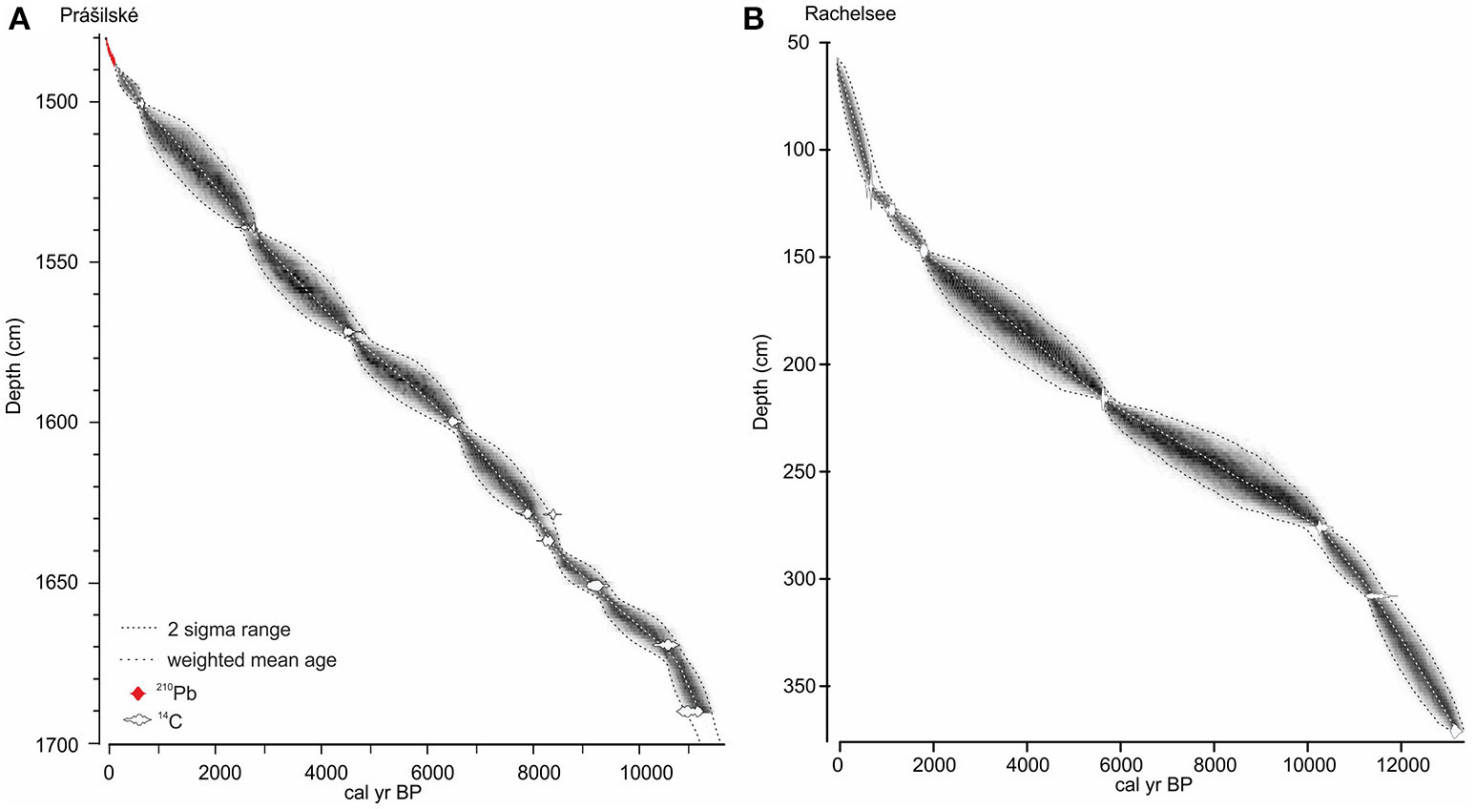
Age-depth models for **(A)** Prášilské jezero, and **(B)** Rachelsee. Models were constructed using BACON. The weighted mean modeled ages against depth were smoothed using a 21-point moving average.

A 2.18 m sediment profile was collected in August 2015 from the deepest (14.8 m) part of Prášilské jezero (49° 04′ N, 13° 24′ E, 1,079 m a.s.l.) and is comprised of two parallel and overlapping cores (PRA 15-2-1 and PRA 15-2-2). The sediment profile was sampled from a floating platform using a hand-percussion Russian-style corer (1.5 × 0.075 m). The sediment–water interface was collected using a 0.1 m diameter gravity corer (PRA15-2GC) (Boyle, 1995). At Rachelsee (48° 58′ 29” N, 13° 24′ 7” E, 1,071 m a.s.l.), 11.8 m of sediment was collected in August 2012 at 12.5 m water depth using a Livingstone piston corer (Wright, 1967) (RAA1-4). Sediment was not recovered between depths 0–57 cm, therefore, the mud-water interface depth begins at 57 cm. Sediment age-depth relationships were established using ^14^C radiocarbon dating at Prášilské jezero (*n* = 10) and Rachelsee (*n* = 7), with an additional ^210^Pb series (Appleby, 1978) at Prášilské jezero (Table 1). For all sites, the geochronological data including the sediment surface were compiled within the Bayesian routine “BACON” (Blaauw and Christen, 2011). This analysis partitioned both cores into 0.05 m thick sections and estimated the accumulation rates for each segment using a Markov Chain Monte Carlo (MCMC) approach (Christen and Pérez, 2009). The analyses were constrained by a prior model of sediment accumulation rate (a gamma distribution with mean 50-year cm^−1^ and shape 1.5) and its variability (memory, a beta distribution with mean 0.5 and shape 16) for both sites. All ^14^C ages were calibrated and modeled in “BACON” using the IntCal13 curve (Reimer et al., 2013), with a Student-t distribution to account scatter in the ^14^C measurements and to allow for statistical outliers (Blaauw and Christen, 2011). The weighted mean modeled ages against depth were smoothed using a 21-point moving average (Figure 3).

### Pollen analysis

At Prášilské jezero, pollen analysis was conducted at 1–2 cm resolution throughout the core by V. A. Carter. For each sample, 0.5 cm^−3^ was processed using standard pollen procedures (Faegri et al., 1989). At Rachelsee, pollen analysis was conducted at 1 cm resolution for 72–124 cm, 2 cm for 126–184 cm, and 4 cm for 188–540 cm by J. F. N. van Leeuwen. The sediment between depths 57–71 cm was too watery and could not be subsampled for pollen. Here, we present data from depths 72–368 cm. At Rachelsee, a minimum of 300 tree pollen grains, and at Prášilské jezero, a minimum of 500 pollen grains were counted in each sample. A *Lycopodium* tablet with a known amount of spores were added to each sample as an exotic tracer in order to calculate pollen concentration and influx rates (Stockmarr, 1972). Total pollen counts were converted into pollen percentages and plotted using Tilia software (Grimm, 1987) based on the abundance of each pollen type relative to the sum of all terrestrial identified pollen. The pollen profiles were divided into pollen assemblage zones based on optimal splitting using the broken-stick model (Bennett, 1996). Pollen counts were converted to concentrations (grains cm^−3^) in Tilia using the counts of *Lycopodium* tracers and sample sediment volumes (Grimm, 1987). The smoothed Bayesian age-depth model for both sites was used to calculate and plot pollen influx rates from the concentration data using Tilia software.

### REVEALS

The REVEALS model (Sugita, 2007a) was applied to estimate pollen-derived regional vegetation cover during the Holocene. Previous studies from the region (e.g., Abraham et al., 2014) mainly used records from peat bogs to calculate a REVEALS output, with the model showing the robustness of peat sequences in portraying present-day vegetation in a realistic manner. Here, the REVEALS model was used to estimate land-cover abundance from both lakes and non-lakes (i.e., peat bogs and mires) using new (this paper) and previously published and unpublished pollen data obtained from the Czech Quaternary Pollen Database (Kuneš et al., 2009; Table 2). Pollen counts were aggregated into 500-year intervals for the entire Holocene, and then all available sites in each 500-year time window were used to estimate mean vegetation abundances within a 60 km radius. The following parameters were selected: 4 m s^−1^ wind speed, different dispersal-deposition models for bogs and mires (Prentice, 1985) and lakes (Sugita, 1994), and taxon-specific relative pollen productivities for 28 selected pollen-equivalent taxa (see Supplementary Table 1; Abraham and Kozáková, 2012; Mazier et al., 2012; Abraham et al., 2014). For model calculations, we adjusted the basin sizes of non-lakes to 4.5 ha. Larger basin sizes produce unrealistic estimates for some taxa as some trees may still grow within the basin, violating the model's assumptions (Abraham et al., 2014). Therefore, assuming smaller non-lake basins was more appropriate for our calculations. Openness was calculated by the summation of all herbs included in the model. All calculations were conducted in R (R Core Team, 2016) using functions to calculate mean vegetation abundances and their error estimates based on bootstrap methods (https://github.com/petrkunes/LRA).

**Table 2:** Regional sites uded to create the REVEALS pollen-based vegetation model.

**Site name**	**Lake or Mire**	**Latitude**	**Longitude**	**Area of site (ha)**	**Adjusted area of site for REVEALS**	**Elevation (m.a.s.l.)**	**Citation**
Černé jezero^*^	Lake	49.18035	13.18538	18.5	18.5	1,008	Unpublished data^*^
Chalupská slat'	Mire	49.00061	13.66286	49	4.5	906	Unpublished data
Finsterauer Filz	Mire	48.948127	13.57751	7.6	4.5	1,055	Stalling, 1987
Heidemühle (Beerenfilz)	Mire	48.826771	13.753396	17	4.5	835	Stalling, 1987
Hurecká slat'	Mire	49.15222	13.32755	62.2	4.5	870	Svobodová et al., 2002
Malá Niva	Mire	48.91376	13.81606	65	4.5	754	Svobodová et al., 2002
Mrtvý luh	Mire	48.8668	13.88292	250	4.5	737	Svobodová et al., 2001
Plešné jezero	Lake	48.77674	13.86571	7.5	7.5	1,105	Jankovská, 2006
Prášilské jezero	Lake	49.07519	13.39976	3.7	3.7	1,080	This study
Rachelsee	Lake	48.974945	13.4019381	5.7	5.7	1,071	This study
Rokytecká slat'	Mire	49.0153	13.4122	200	4.5	1,097	Svobodová et al., 2002
Rybárenská slat'	Mire	49.03129	13.46181	32	4.5	1,014	Svobodová et al., 2002
Soumarské rašeliniště	Mire	48.9066019	13.8388078	30	4.5	750	Svobodová et al., 2001
Stráženská slat'	Mire	48.89887	13.74226	120	4.5	804	Svobodová et al., 2001

Sites with a “^*^” symbol indicate new, but unpublished data that was used to refine the REVEALS model (see Supplementary Material).

## Results

### Regional vegetation development

Müller (1927) originally presented data for 12 tree taxa (Figure 2), however, the discussion of this research will focus on the three dominant canopy species growing in the Bohemian/Bavarian Forests at modern times; spruce (*Picea abies*), beech (*Fagus sylvatica*), and fir (*Abies alba*). Based on the broken-stick model (Bennett, 1996), the pollen percentages profiles from Prášilské jezero and Rachelsee were divided into seven and eight pollen assemblage zones, but for simplicity, the pollen reconstructions were grouped into three main periods; (1) Early- to Mid-Holocene, (2) Mid- to Late-Holocene, and (3) Last-Millennium.

### Early- to mid-holocene (12,000–~6,800 cal. years BP)

**Figure 4:**
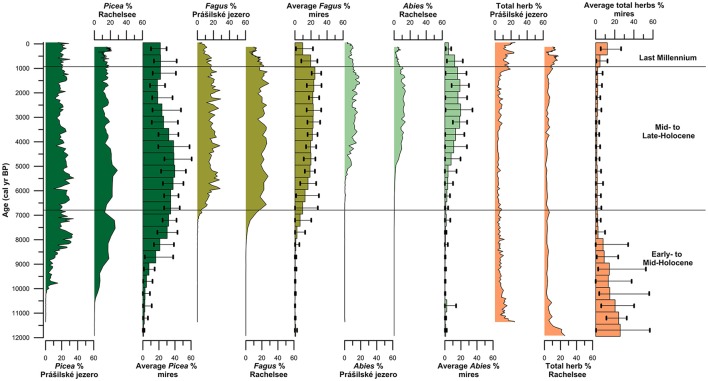
Pollen percentages of the three dominant canopy species (*Picea, Fagus*, and *Abies*) and total herb percentages from Prášilské jezero and Rachelsee, as well as the average percentages and maximum/minimum percentages of all peat bog and mire sites grouped into 500 year bins.

**Figure 5:**
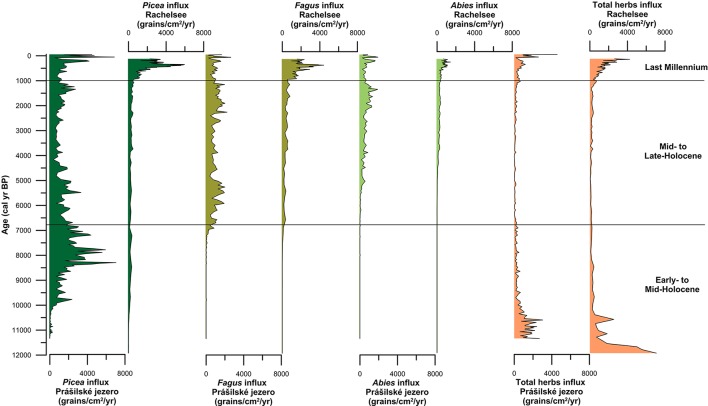
Pollen influxes for the three dominant canopy species (*Picea, Fagus*, and *Abies*) and total herbs from Prášilské jezero and Rachelsee located in the Bohemian/Bavarian Forests of central Europe.

**Figure 6:**
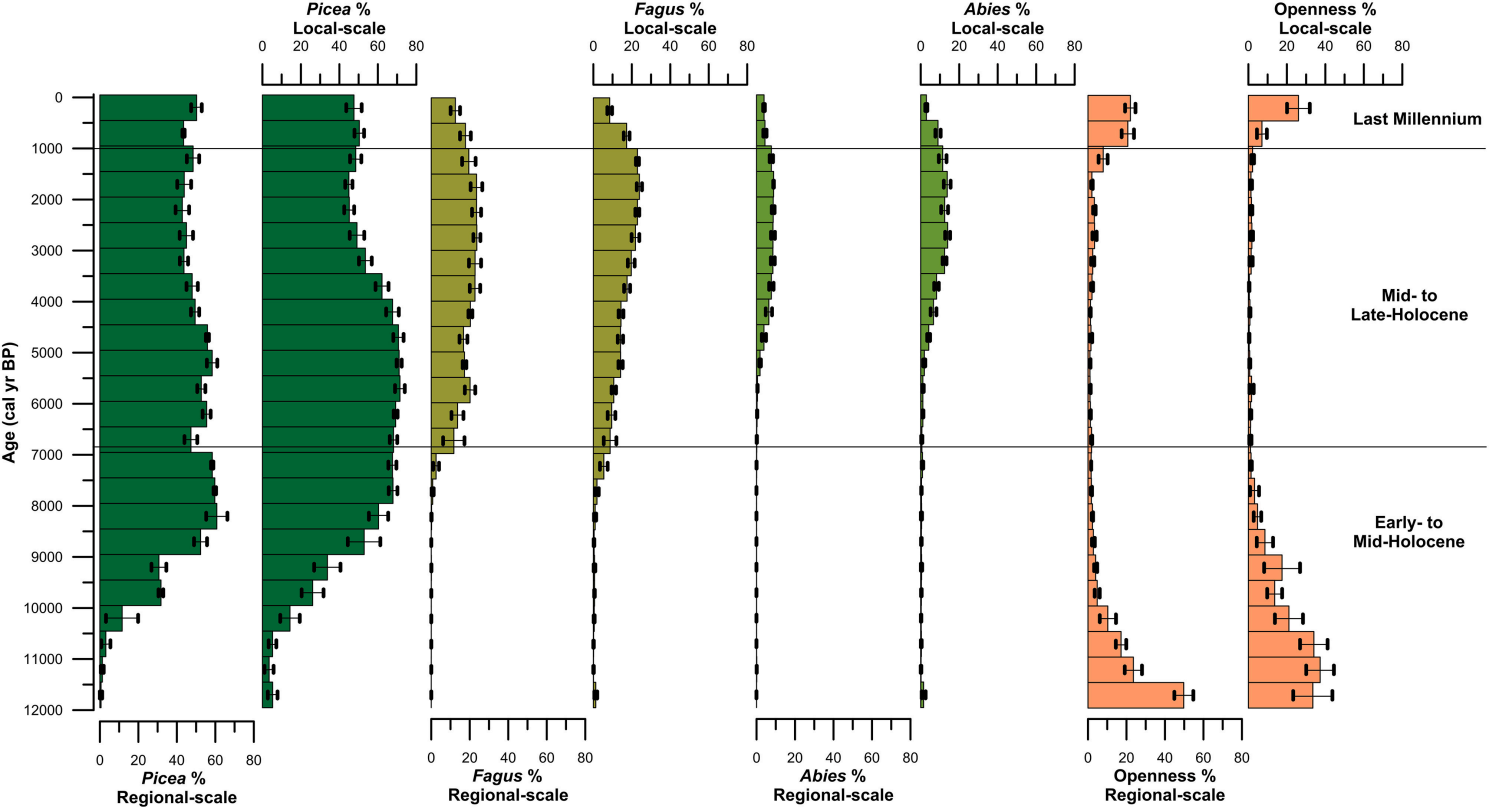
REVEALS estimates of the three dominant canopy species (*Picea, Fagus*, and *Abies*) and total herbs from both lake sites and peat bog/hollow sites located in the Bohemian/Bavarian Forests of central Europe.

Spruce first arrived at the high-elevation lakes Prášilské jezero and Rachelsee between 10,500 and 10,000 years ago, but was present at mid-elevation peat bogs/mires by ~11,500 cal. years BP (Figure 4). Spruce rises to dominance between 10,000–8,500 cal. years BP across all elevations (Figure 4) replacing pine (*Pinus*) and hazel (*Corylus*) as the dominant canopy species (see Supplementary Figures 3–5). Thereafter, spruce percentages fluctuated between 35% and >20% pollen at both Prášilské jezero and Rachelsee, and around 30% at mid-elevation peat bogs/mires, respectively. Pollen influx data for spruce conversely differ between the two sites with a peak of 4,000 grains cm^−2^ yr^−1^ at Prášilské jezero, whereas the increases at Rachelsee are more subdued at <500 grains cm^−2^ yr^−1^ (Figure 5). Quantitative land-cover reconstructions of spruce percentages calculated using REVEALS (Sugita, 2007a) rise around 10,000 cal. years BP from ~5% to >60% calculated on a local (using peat bogs/mire records) basis (Figure 6). On a regional basis (using lake records), spruce comprised >50% total land-cover. The REVEALS land-cover reconstructions suggest that spruce forms close to double the land-cover percentages compared to pollen percentages. Beech begins to expand at both sites from 7,000 cal. years BP onwards, but is at low percentages in the Early- to Mid-Holocene. The dynamics of fir are restricted to after 5,000 cal. years BP. Total herb percentages decrease from ~20% and oscillate around 10% at both Prášilské jezero and Rachelsee. Pollen influx data for total herbs decrease ~10,500 cal. years BP from values >2,000 grains cm^−2^ yr^−1^. The REVEALS reconstruction also suggests that the percent of landscape openness is close to double that of pollen percentage data at both the regional and local-scale (Figure 6).

### Mid- to late-holocene (~6,800–1,000 cal. years BP)

Between 6,800 and 6,000 cal. years BP beech increases gradually in pollen percentages at both Prášilské jezero and Rachelsee, and then oscillates around 25% (Figure 4). Beech percentages from mid-elevation peat bogs/mires begin to increase ~500 years earlier than in the high-elevation lakes. At the local-scale (peat bogs/mires), beech percentages gradually increase throughout the Mid- to Late-Holocene peaking at ~25% roughly 1,000 cal. years BP. Fir expansion commences 5,500–5,000 cal. years BP to around 20% at both Prášilské jezero and Rachelsee. These increases in pollen percentages are associated with no substantial reduction in spruce pollen percentages at Prášilské jezero and Rachelsee, but spruce percentages decrease from ~40% to ~20% <900 m a.s.l. (Figure 4). Viewed from a perspective of pollen influx, the down core pattern of increase and stability in species percentage data are reflected in the influx data (Figure 5). Though as with spruce, influx rates for beech and fir are much lower at Rachelsee. The REVEALS land-cover reconstructions for spruce and fir are lower at the regional scale (i.e., lake sites) relative to the more local-scale (i.e., peat bog/mire sequences), whereas beech is an equally abundant component in both reconstructions comprising ~25% land-cover (Figure 6). The land-cover contribution of spruce remains double (>40%) the values indicated by pollen percentage data (>15%), but these differences are not present for beech and fir. Land-cover percentages of spruce remained relatively stable at the lake sites however, spruce decreases from >60% to ~40% at mid-elevation peat bog/mire sequences during this period. The REVEALS reconstruction also suggests that the percent of openness is <5% at both the regional and local-scales (Figure 6).

### Last-millennium (1,000 cal. years BP-present)

At both lakes, spruce percentages remain unchanged initially and then increase ~5% in the last 300 years (Figure 4). Spruce percentages remain unchanged at mid-elevation peat bogs/mires. Both beech and fir pollen percentages decline at both lakes and mires sequences through the last 1,000 years (Figure 4). Openness percentages increased dramatically over the past 1,000 years to ~12% (Figure 4). Pollen influx rates for all three taxa increase during the last 1,000 years at both lakes, and sharply in the last 300 years which likely reflects focusing of pollen toward the lake center (i.e., in-lake lateral redistribution of materials toward the deeper waters) rather than any change in species abundance (Figure 5). The REVEALS reconstructions broadly confirm the indications seen in the percentage pollen data, with declines in beech and fir occupied by open-ground indicators rather than any expansion in spruce. This is also shown in the pollen influx data, showing a dramatic rise in the influx of open-ground taxa (Figure 5).

## Discussion

### Developments in understanding dominant ecology of the bohemian/bavarian forests

Over the past 100 years, methodological improvements in the field of paleoecology such as radiocarbon dating and pollen-based quantitative land-cover modeling (i.e., REVEALS; Sugita, 2007a) have led to more realistic reconstructions of past vegetation change across temporal and spatial scales. Applying these approaches to the palaeoenvironmental records from Prášilské jezero and Rachelsee illustrates the long-term presence and dominance of spruce in the Bohemian/Bavarian Forests. First appearing in the pollen record ~10,000 cal. years BP, the local presence of spruce in the region is supported by *Picea* stomata at Prášilské jezero and Rachelsee, which agrees with previous paleoecological research in the region (Müller, 1927; Svobodová et al., 2001, 2002; Jankovská, 2006; Abraham et al., 2016). At the regional scale (i.e., lake sites), the REVEALS model suggests that spruce has comprised an average of ~50% of the total forest canopy, whereas at the more local scale (i.e., peat bogs/mires) spruce has comprised ~59%. Thus, spruce has been a dominant component of the forest canopy across a range of elevations >700 m a.s.l. in the Bohemian/Bavarian Forests.

Modern quantitative approaches also illustrate a unique history of forest composition throughout the region with beech and fir contributing as secondary canopy species relative to spruce at mid-elevations found between 700 and 900 m a.s.l. Beech established around 7,000 cal yr BP and became an abundant canopy species around 6,000 cal. years BP, with the REVEALS model indicating that it contributed ~20% of the total land-cover at the regional scale. However, at the more local-scale between 700 and 900 m a.s.l. beech comprised just 16% of the total land-cover during the Mid-Holocene. After the establishment of beech, Müller (1927) found an increase in fir percentages. Svobodová et al. (2001, 2002) suggested that fir had spread to elevations >700 m a.s.l. across the entire region between 6,300 and 3,400 cal. years BP. However, these records were constrained by few radiocarbon dates. At Prášilské jezero and Rachelsee, fir first appeared between 7,000–6,300 cal. years BP but did not become an important component of the canopy until 5,000–4,500 cal. years BP. The REVEALS model output indicates that for the last 4,000 years, fir has comprised an average of ~7% of the total land-cover at the regional-scale, but slightly higher (10%) at the more local-scale between 700 and 900 m a.s.l. As beech and fir expanded around 4,000 cal. years BP, spruce percentages declined to some extent but still comprised ~40% of the regional total land-cover, and >60% of the local-scale (i.e., mid-elevation) forest composition in the Bohemian/Bavarian Forests (Figure 6). This further demonstrates that beech has been a secondary canopy species relative to spruce at mid-elevations, specifically between 700 and 900 m a.s.l. Total land-cover of beech and fir populations found at mid-elevations began to decrease from their maximum values of 24% and 13% total land-cover ~1,000 years ago at both the regional and more local-scale (Figure 6). The decrease in beech and fir forests are likely the result of increasing anthropogenic landscape modifications. However, climate could also explain the decline in beech and fir as temperatures were likely unfavorable for these species during the Little Ice Age (Grove, 2001; Brázdil et al., 2017).

Declines in beech and fir characterize the last millennium with an expansion in open ground taxa in both the percentage pollen data and the quantitative REVEALS land-cover reconstructions, though spruce is slightly more abundant in terms of total land-cover (Figure 6). Svobodová et al. (2002) also indicate that beech-spruce-fir forests have decreased over the past three centuries, and are being replaced by spruce and *Pinus rotundata* in forest bogs at lower elevations. The regional reconstructions presented here point to a change in forest composition toward more open ground within the forest, and slightly increased dominance by spruce. Over the past 500 years spruce has comprised ~47% land-cover, while beech and fir has comprised ~8% and <5% at mid-elevations. The pollen influx values show a radically different story with the records from Prášilské jezero and Rachelsee illustrating an increase in all pollen taxa through the last 500 years. This increase in pollen influx likely reflects increased in-lake focusing of pollen to the lake center assisted by wind currents from a more open landscape (see Figure 5 for percent openness), rather than increased pollen rain from spruce plantations found at low-elevations. Openings within high-elevation forests are generally attributed to human clearance, fire, windthrow, or bark beetle outbreaks (*Ips typographus*) which are key disturbance drivers influencing Norway spruce forest dynamics (Holeksa and Cybulski, 2001; Fischer et al., 2002; Holeksa et al., 2007; Svoboda et al., 2012, 2014; Čada et al., 2016; Janda et al., 2017; Kulakowski, 2017). During the 1990s, an *I. typographus* bark beetle outbreak occurred in the region creating gaps (i.e., openings) in the canopy. Severe windstorms occurred in the region in 1986, 1999, 2007, and 2008, which also caused widespread forest damage (Svoboda et al., 2010). Yet, Zeppenfeld et al. (2015) found that spruce seedlings and young spruce trees already occur in areas affected by these large-scale disturbances, encouraging rapid recovery of spruce and not limiting flux of pollen to the lakes in the region. Therefore, increase in pollen fluxes especially at Prášilské jezero is likely a result of natural processes and not a result of spruce plantations found at lower elevations.

While the REVEALS model demonstrates that spruce has been the dominant canopy cover at both high- and mid-elevations, the model only provides an average percentage of land-cover for the entire Bohemian/Bavarian Forest. Local site conditions such as slope and aspect affect forest composition at the more local-scale (i.e., stand-scale). For example, spruce generally dominates on north-facing mesic sites with poorly-drained soils, whereas beech typically dominates on south-facing xeric sites where soils drain well. These local conditions can lead to stands of monospecific spruce or beech at mid-elevations, specifically in the Bavarian Forest National Park. However, the REVEALS model used in this study was used to inform managers of the natural average forest composition for elevations >700 m a.sl. for the area through time.

### Applications to conservation and restoration in the bohemian/bavarian forests

The overall management goal of the parks is to restore the original forest composition (IUCN, 1994). Within the Šumava National Park forest management has likely based their target “natural” forest composition from Geobotanical Maps (Mikyška et al., 1968–1972), and the Map of Potential Natural Vegetation (Neuhäuslová et al., 1998), which suggest that beech dominate over spruce at mid-elevations in central Europe (Ellenberg and Leuschner, 1996). More recently, Vacek and Mayová, 2000, suggest that beech is dominant over spruce at mid-elevations between 650 and 900 m. Together, these previous maps and research have influenced the management strategy of removing spruce from mid-elevation forests within Šumava National Park so that spruce will comprise 30–40% instead of its current representation of “84%” (Šumava National Park Authority^2^). However, these maps and previous research do not consider paleoecological studies which provide long-term dynamics of vegetation abundance through time. Paleoecological studies have utility in conservation and restoration in that they can provide baseline targets (i.e., more accurate representations of forest composition), as well as provide the natural range of variability within vegetation composition through time. While the REVEALS model shows that beech and fir forests reached a combined maximum of 37% at mid-elevations (between 700 and 900m a.s.l.) ~2,000 years ago, spruce forests still comprised 45% of the total forest composition at these elevations (Figure 6). A regional paleoecological perspective of the past 500 years shows that the forest composition at mid-elevations has been a mixed-forest cover of spruce (~50%), beech (~22%), and fir (~10%), which counters the findings of Vacek and Mayová (2000) Our findings support previous investigations that highlight that mixed-forests consisting of spruce-beech-fir typically occur between 650 and 1,150 m in the Šumava National Park (Röder et al., 2010), as well as showing the long-term dominance of coniferous forests in the Bohemian-Moravian highlands over the past 9,000 years (Szabó et al., 2017). The long-term presence of spruce throughout the Holocene illustrates that forest management practices involving the formation of spruce plantations at mid-elevations have not suppressed beech (Szabó et al., 2017). During the late Holocene the results from the regional-scale sites show some suppression of beech (~15% land-cover) that is not related to spruce dominance, but rather to the extent of open ground. This supports previous research from Šumava National Park with beech rarely reaching >20% from mid-elevation sites (Hrubý et al., 2014).

Within the Bavarian Forest National Park, soil conditions and south-facing slopes create more favorable conditions for beech and fir forests. While spruce has doubled its range since the nineteenth century in the region, foresters have been more concerned with the recent decline of fir. At the time of establishment of the Bavarian Forest National Park, fir comprised only 3.2% of the total forest composition (Heurich and Englmaier, 2010). This is supported by the regional-scale REVEALS reconstruction which shows the decline of fir over the past 500 years to 3.8%. However, at the more local-scale (i.e., peat bogs/mires) at mid-elevations fir has declined to just 2.9% (Figure 6). Stalling (1987) found that fir pollen frequencies reached a peak of ~32% fir roughly 3,000 years ago on the basis of 15 sites with a mean elevation of 837 m a.s.l. in the Bavarian Forest. However, quantitative land-cover reconstructions presented here indicate that fir reached a maximum regional land-cover of just 13% between 3,500 and 1,000 cal. years BP, and a maximum of ~20% at the more local-scale at mid-elevations (Figure 6). Methodological improvements such as quantitative land-cover modeling offer a more robust reconstruction of vegetation abundance from the Bohemian/Bavarian Forests of central Europe. Thus, the results presented here provide a more accurate and updated description of fir abundances than the previous work by Stalling (1987). However, the REVEALS model is limited in that all of the mid-elevation sites used in the model are located within the park boundaries of Šumava National Park. Mid-elevation paleoecological reconstructions do exist from the Bavarian Forest National Park, yet, the sites were not incorporated into the model because they were either not available publicly or they lacked radiocarbon dating. Therefore, the results interpreted here may be more reflective of vegetation abundances from mid-elevation in Šumava National Park. Regardless, the REVEALS model reconstructed the regional decline of fir, and it is therefore a concern that should be addressed by foresters in the Bavarian Forests. Heurich and Englmaier (2010) suggest that the trend of declining fir populations relative to increasing spruce populations in the Bavarian Forests is likely not solely related to human activity but also to natural causes such as the Little Ice Age and bark beetle outbreaks. Because spruce is a cold adapted species, conditions during the Little Ice Age would have favored spruce over fir. Additionally, downed logs from windthrow and bark beetle outbreaks are the preferred natural substrate that gives spruce seedlings a competitive advantage over other tree species (Svoboda et al., 2010). While several studies suggest that fir is sensitive to human activities and anthropogenically caused air pollution (Tinner et al., 1999; van der Knaap et al., 2004; Tinner and Lotter, 2005; Feurdean and Willis, 2008), human impacts in the form of pasturing, selective logging and litter raking have been shown to be beneficial for fir growth (Nožička, 1957; Málek, 1981; Kozáková et al., 2011). Tinner et al. (2013) suggest that the range of fir has the potential to increase with climate change as long as precipitation does not decrease below 700–800 mm/yr, or anthropogenic disturbances such as fire and grazing do not become excessive.

One of the benefits of the Šumava and Bavarian Forest National Parks is that it serves to protect montane spruce forests (Svoboda et al., 2010). Spruce is likely to be the species most impacted by climate change, and so is under threat in a warmer world (Bolte et al., 2009). Norway spruce is considered a cold-adapted species growing on mesic sites. Therefore, expected changes in temperature and precipitation in the future specifically the increased likelihood of more frequent and intense summer droughts across central and southern Europe (Gao and Giorgi, 2008; Feyen and Dankers, 2009), will likely result in the loss of available habitat for spruce (Hanewinkel et al., 2012). In the Bohemian/Bavarian Forests, Norway spruce has already occupied the highest possible altitudes and ostensibly the species has nowhere to go with climate change (Spathef et al., 2014). Conversely, beech and fir forests, the main competitors of spruce in the region, could continue to impinge on and expand in the lower altitude habitat of spruce. However, areas where beech predominates likely will also be threatened with increasing temperatures (Cheddadi et al., 2016). Migration of the optimum climate windows for these dominant forest taxa is a concern for forest managers, yet our paleoecological data show these plant communities have comprised a mixed-forest for >5,000 years in the region surviving previous climatic fluctuations during the Holocene. Mixed-forests with multiple tree species provide higher levels of ecosystem services (Gamfeldt et al., 2013) and should therefore be incorporated into forest management in the Bohemian Forests. We suggest that forest managers in the Šumava National Park consider utilizing information gained by paleoecological reconstructions that use pollen-based land-cover models that more accurately represent the natural vegetation abundances found at mid-elevations.

## Conclusions

Methodological advances in the field of palynology and paleoecology, including quantitative land-cover models and robust chronologies have allowed for more accurate reconstructions of vegetation dynamics through time. Using these methodologies the results of this study demonstrate that spruce has been the dominant canopy cover in the Bohemian/Bavarian Forests for the past 9,000 years across a range of elevations including mid-elevations (between 700 and 900 m a.s.l.) where beech and fir forests were previously thought to dominant. When beech and fir forests peaked around 2,000 years ago, together they comprised a total land-cover of 37%. However, spruce contributed 40% of the total land-cover, further documenting the dominance of this species in mixed beech/fir forests. Over the past 500 years, spruce has comprised ~47% land-cover, while beech and fir comprised ~8% and <5% at mid-elevations.

Paleoecological results have considerable utility in forest management and ecosystem conservation (e.g., Birks, 1996; Jackson, 1997; Landres et al., 1999; Swetnam et al., 1999; Foster et al., 2003; Gillson and Willis, 2004). For example, forest management in the Šumava National Park base current management strategies on outdated information that states that beech and fir forests are the natural, dominant vegetation at mid-elevations. However, the results presented here demonstrate that mid-elevations forests have been a mixed-forest with spruce dominating the forest canopy. The current management plan is to remove spruce to a natural representation of 30–40%, while increasing beech and fir up to a total of 35% (Šumava National Park Authority^2^). The longer perspective afforded by paleoecology suggests that the Šumava National Park should aim for 45% spruce as that is within the natural range of total land-cover for the species, but ecosystem managers should take into account the potential for climate change to significantly impact the range of spruce within the park and take steps toward preserving the species. As for the Bavarian Forest National Park, the REVEALS model was able to capture a decline of fir cover to levels previously recorded (see Heurich and Englmaier, 2010), yet the results may be more indicative of vegetation abundances within Šumava National Park. Additional paleoecological sites with robust chronologies are needed from the region, specifically from between 400 and 700 m a.s.l. in order to compare local-scale vegetation dynamics through time.

## Author contributions

PK and JC: obtained funding to support the project; PK, JC, RC, and AM: collected the data; PK, JvL, RC, and VC: analyzed the data; VC and PK: conceived ideas and led the manuscript writing; VC, RC, JC, NK, AM, MS, HS-S, JvL, and WvdK: edited the manuscript during all phases.

### Conflict of interest statement

The authors declare that the research was conducted in the absence of any commercial or financial relationships that could be construed as a potential conflict of interest.

## Footnotes

^1^Bavarian Forest National Park Authority: http://www.nationalpark-bayerischer-wald.de/english/nationalpark/management/index.htm.

^2^Šumava National Park Authority. http://www.npsumava.cz/en/3291/sekce/the-species-composition-of-the-forests/.
