# Supplementary material for: Quantitative Palynology Informing Conservation Ecology in the Bohemian/Bavarian Forests of Central Europe
Source: Front Plant Sci. 2018 Jan 17;8:2268. doi: 10.3389/fpls.2017.02268 (PMC5776123; doi:10.3389/fpls.2017.02268)

Supplementary Material

Quantitative palynology informing conservation ecology in the Bohemian/Bavarian forests of central Europe

Vachel A. Carter*, Richard C. Chiverrell, Jennifer L. Clear, Niina Kuosmanen, Alice Moravcová, Miroslav Svoboda, Helena Svobodová-Svitavská, Jacqueline F.N. van Leeuwen, W.O. van der Knaap, Petr Kuneš

*** Correspondence:** Corresponding Author: vachel.carter@gmail.com

# Supplementary Data

In August 2015, on the same coring excursion as Prášilské jezero, a 4.3 m sediment profile from Černé jezero (49° 10.8’ N, 13° 11.1’ E, 1008 m a.s.l.) was collected from a depth of 17.1 m. Sediment age–depth relationships were established using four ^14^C radiocarbon dates (Table SI2), and were calibrated and modelled in ‘BACON’ using the IntCal13 curve (Reimer et al., 2013), with a Student-t distribution to account scatter in the ^14^C measurements and to allow for statistical outliers (Blaauw and Christen, 2011) (Figure SI1).

Černé jezero Holocene sediments were analysed in 10 cm resolution for pollen by P. Kuneš. At Černé jezero, 500 pollen grains were counted in each sample (Figure SI2). Pollen data were incorporated into the REVEALS model in order to make the regional-scale model more robust.

# Supplementary Figures and Tables

**Supplementary Table 1**. Summary of age-depth relationships for Černé jezero, Czech Republic.


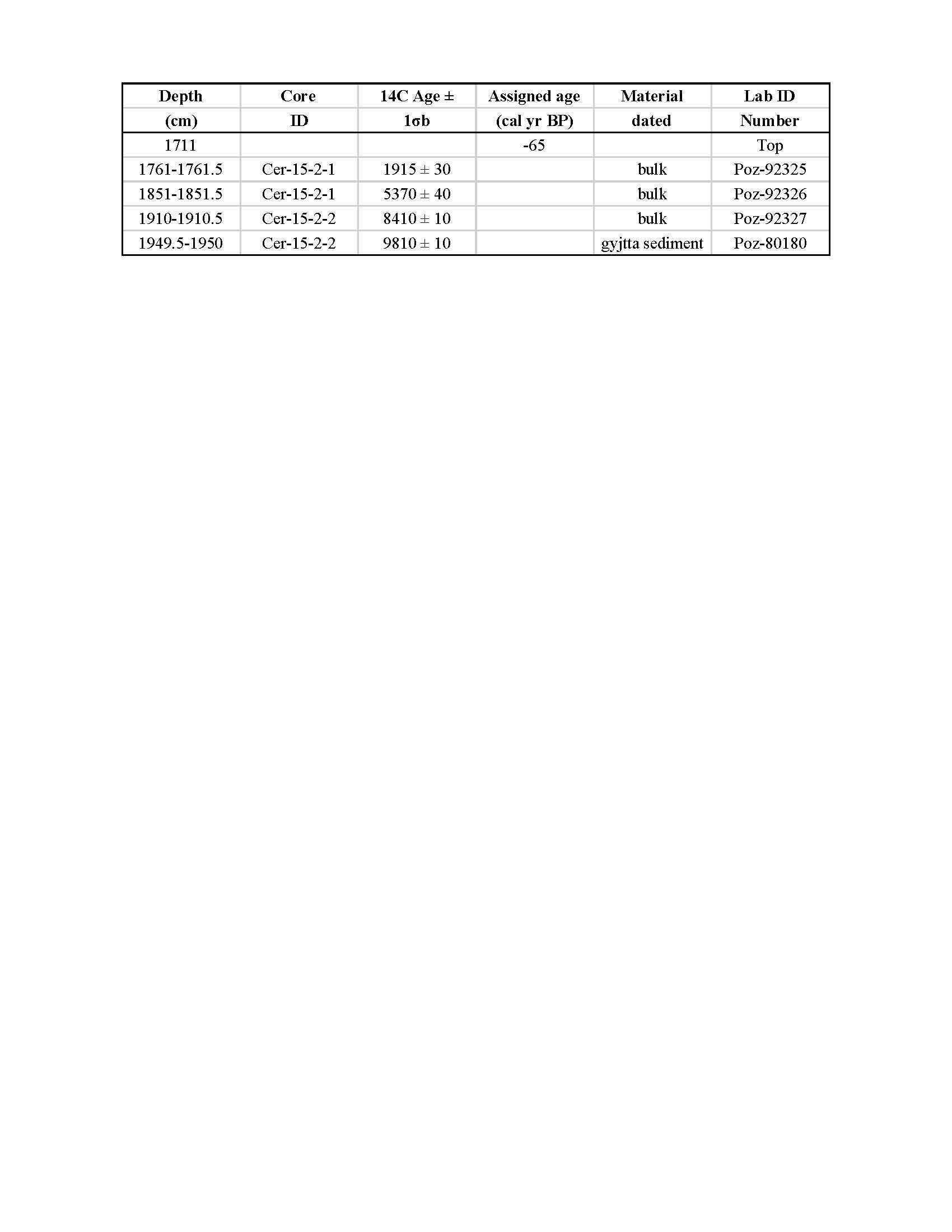


**
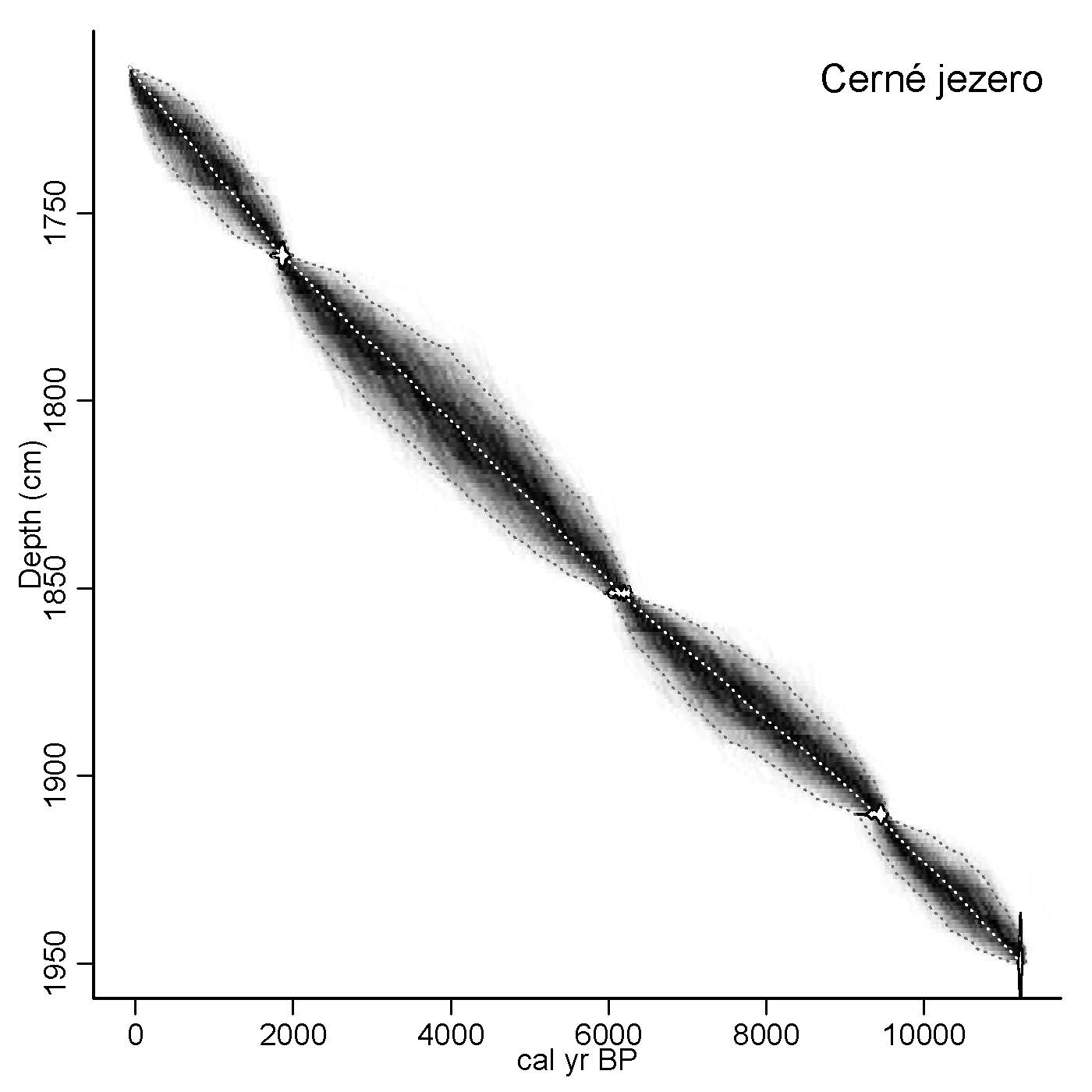
**

**Supplementary Figure 1.** Age-depth model for Černé jezero, Czech Republic. The model was constructed using Bacon.


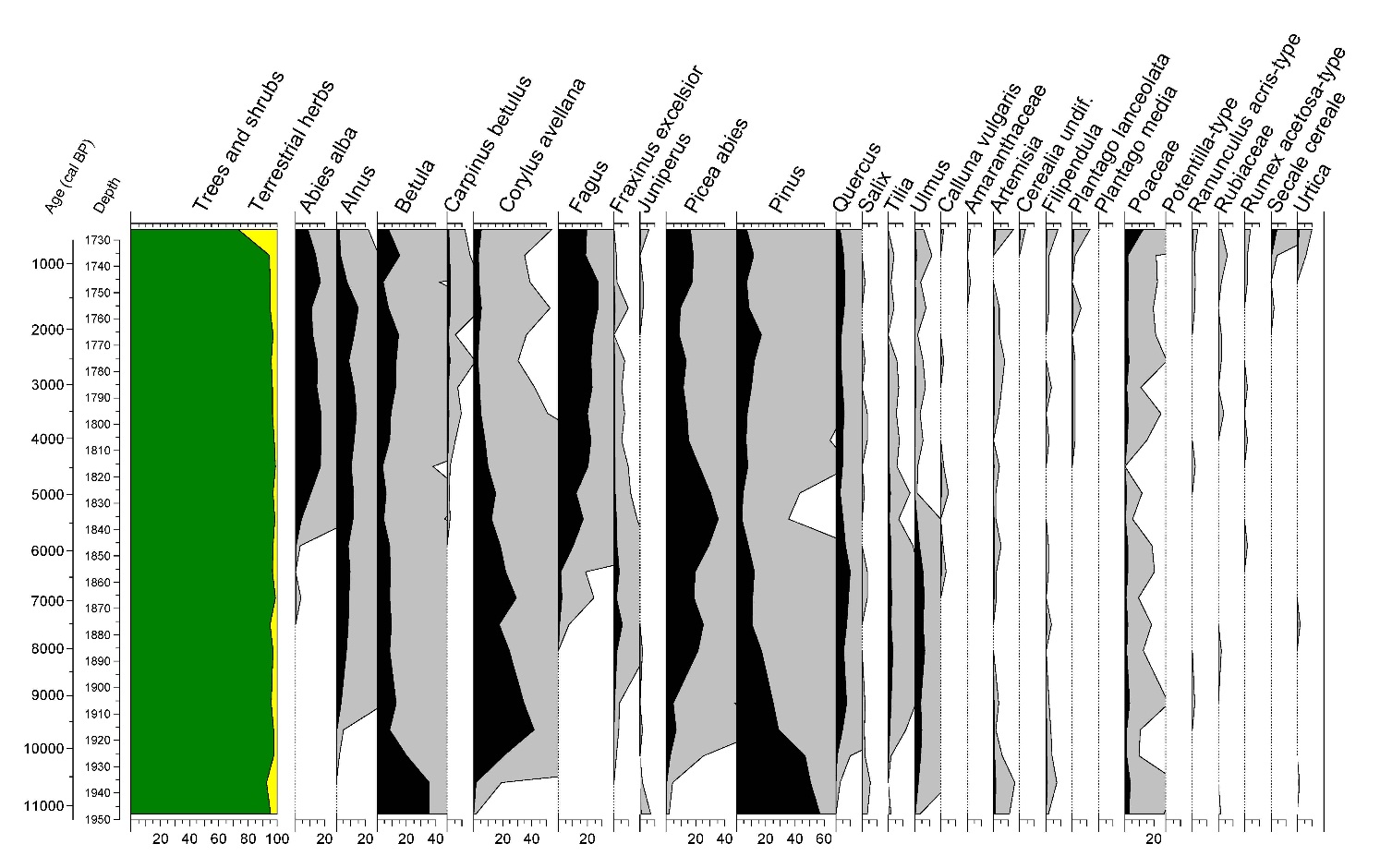


**Supplementary Figure 2.** Pollen percentage diagram for Cerné jezero, Czech Republic. Pollen counts from Černé jezero were included in the REVEALS model in order to calculate regional land-cover abundance.


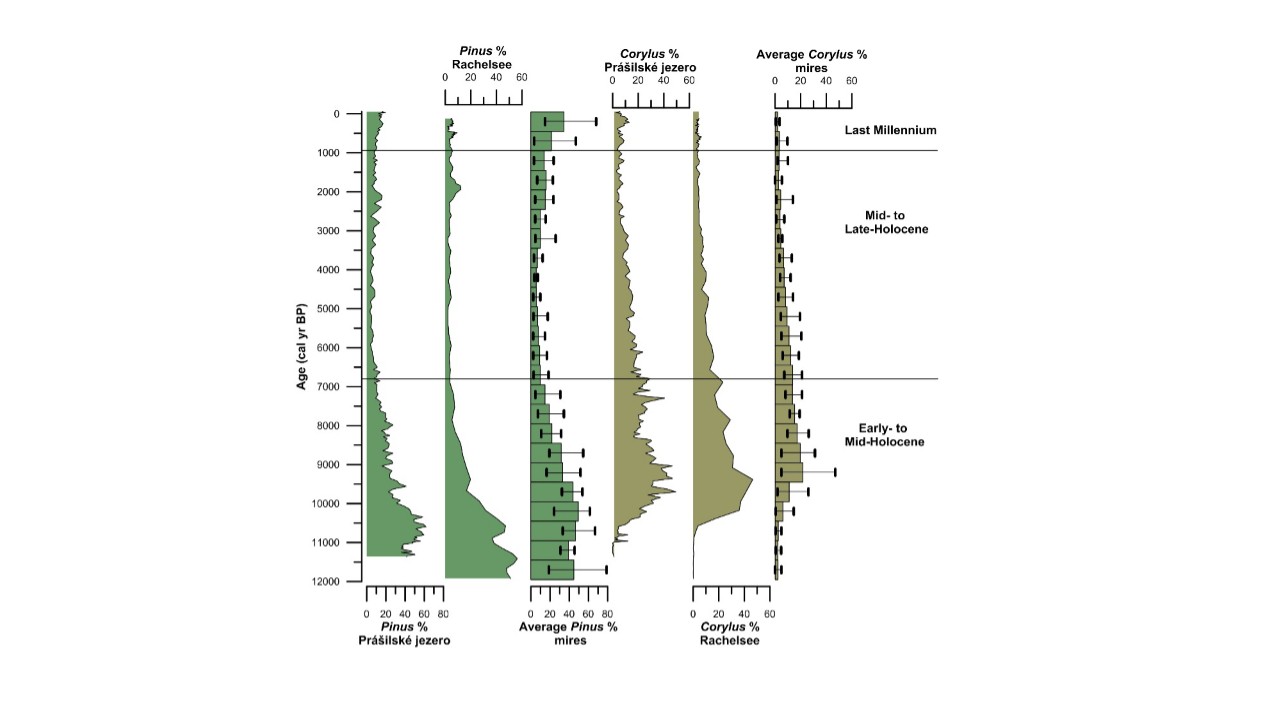


**Supplementary Figure 3.** Pollen percentages of pine (*Pinus*) and hazel (*Corylus*) from Prášilské jezero and Rachelsee, as well as the average percentages and maximum/minimum percentages of all peat bog and mire sites grouped into 500 year bins.


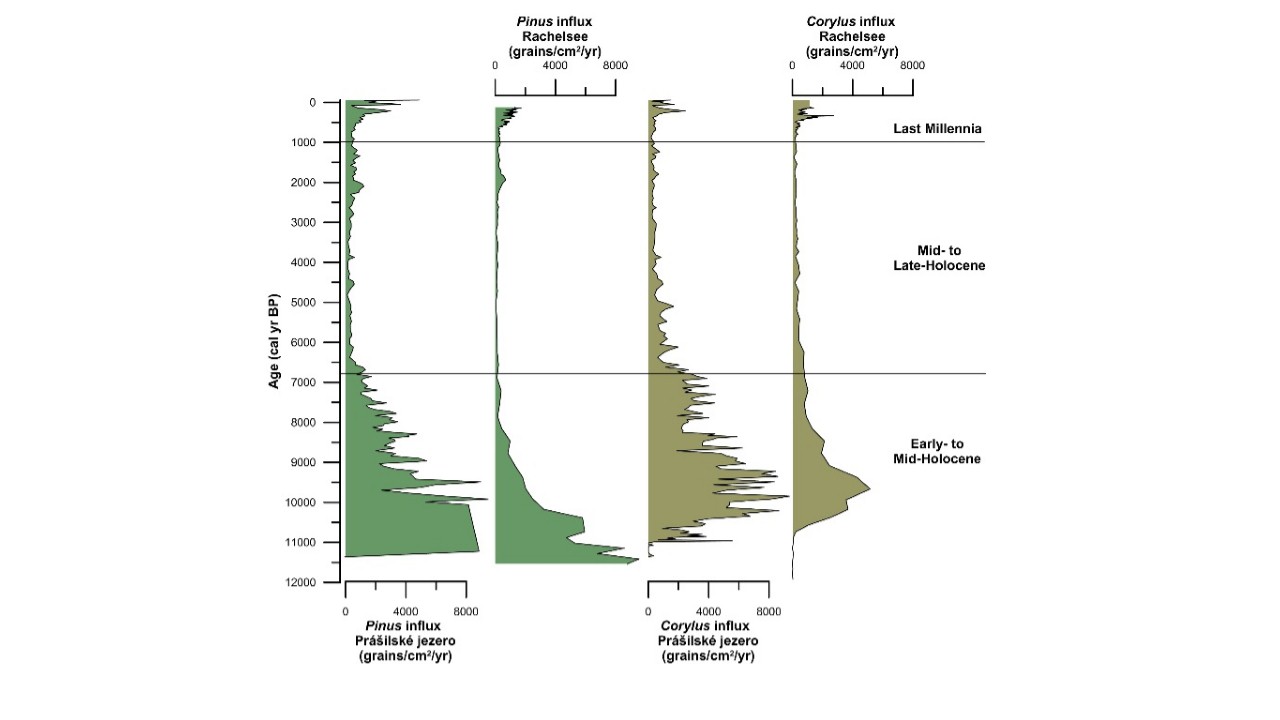


**Supplementary Figure 4.** Pollen influx for pine (*Pinus*) and hazel (*Corylus*) from Prášilské jezero and Rachelsee located in the Bohemian/Bavarian Forest of central Europe.

**
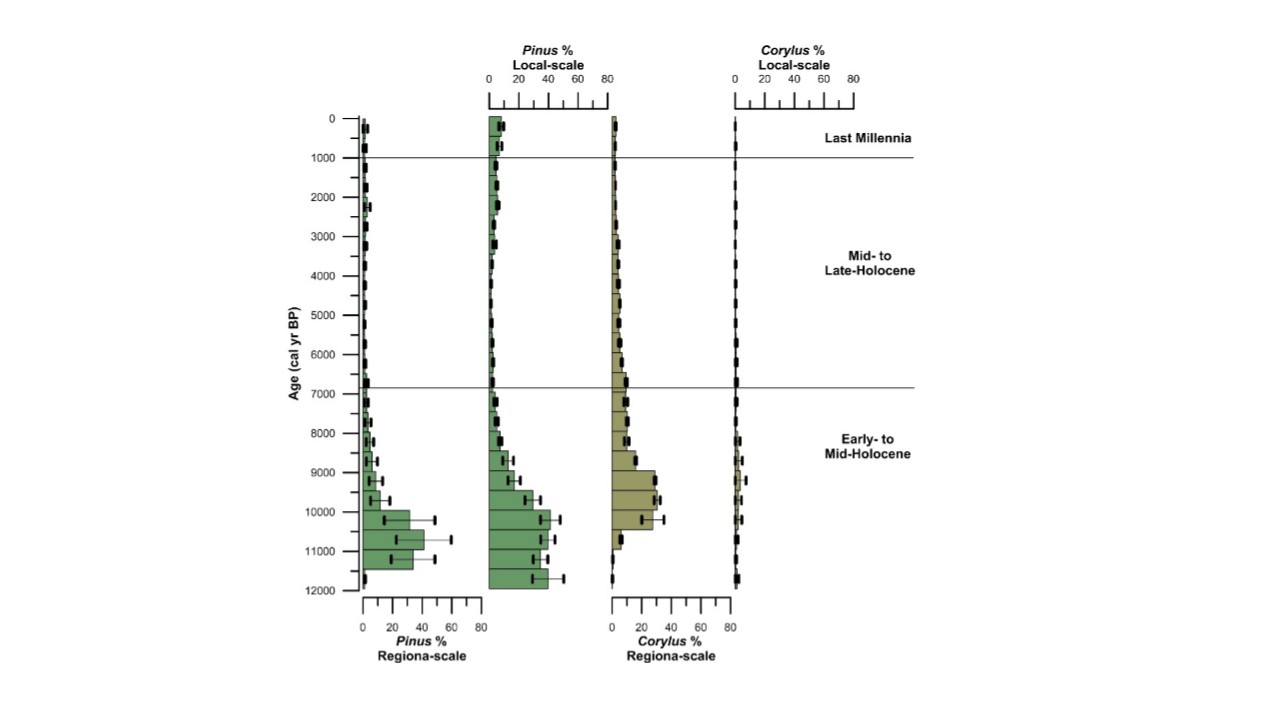
**

**Supplementary Figure 5.** REVEALS estimates for both lakes, and peat bogs/forest hollows from the Bohemian/Bavarian Forest of central Europe.

**Supplementary Table 2.** Selected taxa included in the REVEALS model used to calculate total land-cover abundance.


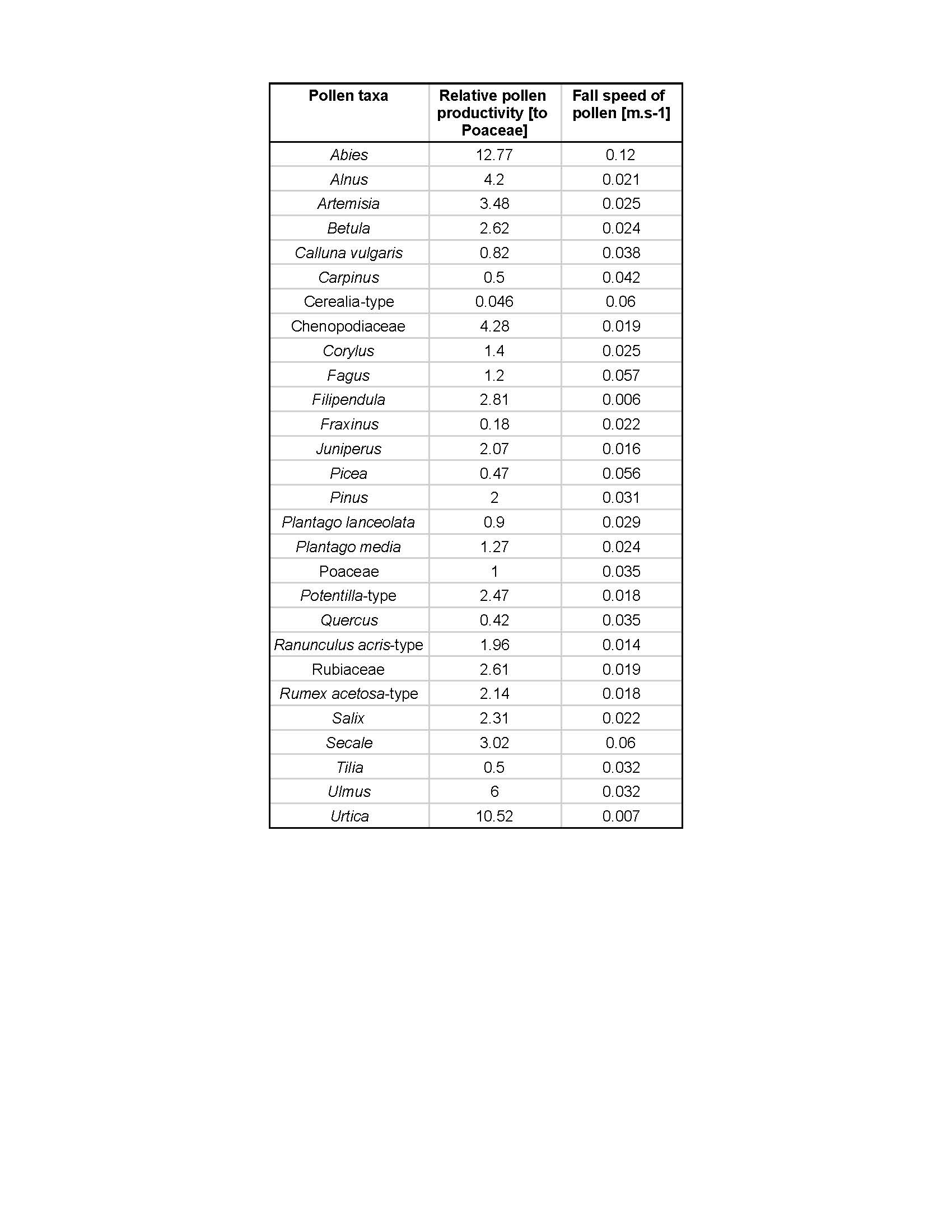

Supplement: Supplementary file 1 [file DataSheet1.DOCX]
